# Remote monitoring to improve engagement for patients with inflammatory bowel disease: a randomized clinical trial

**DOI:** 10.1093/crocol/otag076

**Published:** 2026-07-07

**Authors:** Shivan J Mehta, Phuoc Nguyen, Caitlin Brophy, Catherine Reitz, Christopher K Snider, Meenakshi Bewtra, James D Lewis

**Affiliations:** Division of Gastroenterology and Hepatology, Perelman School of Medicine, University of Pennsylvania, Philadelphia, PA, United States; Population Health Lab, Center for Health Care Transformation & Innovation, University of Pennsylvania, Philadelphia, PA, United States; Division of Gastroenterology and Hepatology, Perelman School of Medicine, University of Pennsylvania, Philadelphia, PA, United States; Population Health Lab, Center for Health Care Transformation & Innovation, University of Pennsylvania, Philadelphia, PA, United States; Division of Gastroenterology and Hepatology, Perelman School of Medicine, University of Pennsylvania, Philadelphia, PA, United States; Population Health Lab, Center for Health Care Transformation & Innovation, University of Pennsylvania, Philadelphia, PA, United States; Division of Gastroenterology and Hepatology, Perelman School of Medicine, University of Pennsylvania, Philadelphia, PA, United States; Population Health Lab, Center for Health Care Transformation & Innovation, University of Pennsylvania, Philadelphia, PA, United States; Population Health Lab, Center for Health Care Transformation & Innovation, University of Pennsylvania, Philadelphia, PA, United States; Division of Gastroenterology and Hepatology, Perelman School of Medicine, University of Pennsylvania, Philadelphia, PA, United States; Division of Gastroenterology and Hepatology, Perelman School of Medicine, University of Pennsylvania, Philadelphia, PA, United States

**Keywords:** inflammatory bowel disease, remote monitoring, behavioral science, text messaging

## Abstract

**Background:**

Biologic therapy has improved care for patients with inflammatory bowel disease (IBD), but adherence is often suboptimal, and a gap in care is lack of engagement between visits. We evaluated the effect of a behaviorally-informed remote text monitoring program on outcomes and adherence among patients with IBD.

**Methods:**

Adults with IBD and receiving biologics through home health or self-injection were individually randomized 1:1 to control (usual care) or intervention (remote monitoring), stratified by medication type (home infusion vs. injection). The 4-month intervention included the following components via text message: (1) biologic dose reminders, (2) medication adherence check-ins after each dose, (3) weekly symptom check-ins with escalation to the patient’s care team if above threshold, and (4) feedback to an identified support partner (when applicable). The primary outcome was change in quality of life via the Short Inflammatory Bowel Disease Questionnaire (SIBDQ).

**Results:**

150 patients were enrolled; 148 completed the study, and 144 were analyzed for the survey outcomes (73 control, 71 intervention). The mean age was 38.9, 52.7% were female, and 81.1% were White. No significant differences were found between arms for the change in median score from baseline to end of study for the SIBDQ, MARS-5, or patient satisfaction surveys. Medication adherence remained high across both arms, and there was no significant difference in the proportion of days covered for infusion or injection between arms.

**Conclusions:**

Remote monitoring was feasible with high patient satisfaction, but there was no improvement in quality of life or medication adherence.

**Trial Registration:**

clinicaltrials.gov NCT04388865

## Introduction

Crohn’s Disease (CD) and Ulcerative Colitis (UC), collectively known as inflammatory bowel disease (IBD), are common chronic gastrointestinal diseases with significant morbidity and decreased quality of life.^1^^,^^2^ Care for patients with IBD has been transformed by biologic therapies which dramatically improve inflammation and clinical outcomes, but also have substantial cost for the health care system.^3–5^ As they are delivered through infusion or subcutaneous injection with long intervals between doses, it can be challenging for patients to maintain adherence, and there is evidence of lower adherence and less engagement with home infusion therapy.^6^ Barriers to adherence include systems issues, such as prior authorization and site of service limitations, and traditional reasons for non-adherence such as inertia and present-time bias. Despite evidence that high adherence is needed for effectiveness, estimates are as low as 66%.^7^^,^^8^

Clinicians typically lack knowledge of how their patients are doing between visits. Specifically, patients may not adhere to necessary therapy, and physicians are unaware of changes in the course of the disease. Patient-reported outcomes are associated with control of the disease, but serially monitoring these measures has not been tested prospectively.^9^ Assessing symptoms weekly is highly correlated with information collected with a more cumbersome daily diary, which may provide additional data to better manage symptoms and therapy.^10^

Remote monitoring using text messaging provides an opportunity to connect with patients between visits.^11^^,^^12^ There are also new tools from the field of behavioral science that can inform such interventions, including nudges that invoke precommitment or reciprocity, including social support from friends or family members, and connecting to clinicians to provide accountability and act upon changes in symptoms.^13^ Remote monitoring programs have shown benefit in areas such as hypertension and joint replacement,^14^^,^^15^ but there is limited evidence of this approach for patients with inflammatory bowel disease.^16–18^

In this randomized clinical trial, we evaluate the effectiveness of a remote monitoring program for patients with IBD that includes symptom monitoring and medication reminders.

## Methods

### Study design

This was a prospective, randomized controlled trial aimed at developing and testing a remote monitoring program called PATH-IBD (Patient Automated Text Hovering for Inflammatory Bowel Disease). Eligible patients were randomized in a 1:1 ratio into one of two arms: (1) control receiving usual care, or (2) intervention receiving remote monitoring of clinical symptoms and medication adherence reminders with social support. The protocol and statistical analysis plan are reported in a Supplement, and the trial was registered on clinicaltrials.gov (NCT04388865).

### Study population

This study was conducted across 4 gastroenterology practices with 17 physicians at an urban academic health system in the Philadelphia region. Eligible patients were 18 years of age or older with a diagnosis of CD or UC, with at least 2 visits with a participating IBD specialist within the past 2 years, currently prescribed a biologic therapy (infliximab, adalimumab, ustekinumab, certolizumab, golimumab, or vedolizumab, including biosimilars), and receiving their biologic treatment through home health infusion and/or self-injection. Patients were excluded if they do not have a phone with text messaging abilities, are non-English speaking requiring a translator, pregnant, or with a known ostomy.

### Recruitment and randomization

Prior to study start, all IBD specialists were informed by email of the study and provided the opportunity for them to opt-out on behalf of their patients. Potentially eligible patients were then identified through automated data extraction from the electronic health record (EHR) in January 2021. Due to an uneven distribution of self-injection and home infusion patients (3:1) identified, we under-sampled the self-injection patients to allow for an even number of home infusion (*N* = 318) and self-injection (*N* = 318) patients in the data sample. The patients (*N* = 636) were then randomly sorted for chart review using a random number generator. After reviewing the chart to confirm eligibility, each patient was mailed a recruitment packet containing an introduction letter, study brochure, informed consent form, and a copy of the baseline surveys. Beginning approximately 1 week after mailing, each patient received up to 5 phone call attempts to complete enrollment. The enrollment phone call included completion of the eligibility survey, verbal informed consent, all baseline survey instruments, randomization, and notification of arm assignment. The baseline survey included the Short Inflammatory Bowel Disease Questionnaire (SIBDQ), Medication Adherence Report Scale (MARS-5), and patient satisfaction survey (including some prompts adapted from the CACHE questionnaire along with additional prompts related to the patient’s perception of quality of care).^19^^,^^20^^,^^21^^,^^22^ Use of the Short Inflammatory Bowel Disease Questionnaire, authored by Dr. Jan Irvine et al., was made under license from McMaster University, Hamilton, Canada.

Randomization was completed using permuted block randomization with random block sizes of 4 and 8 and stratified by medication administration type (self-injection vs home infusion). If the patient was randomized to the intervention arm and provided information for a support partner (not required), up to three phone call attempts were made to complete support partner enrollment. All patients regardless of arm assignment received $25 compensation for completing the enrollment phone call and another $25 for completing the end of study phone call.

Recruitment was completed on a rolling basis from February 23, 2021 through September 12, 2022, and each patient was enrolled in the program for a total of four months with the last enrolled patient completing the program on January 10, 2023.

### Interventions

Patients randomized to the control arm received usual care. Patients randomized to the intervention arm received the PATH-IBD clinical hovering program. The program included the following components all sent via text message: (1) biologic dose reminders, (2) medication adherence check-ins after each dose, (3) weekly symptom check-ins with escalation to the patient’s care team if above threshold, and (4) feedback to the identified support partner (when applicable). Remote monitoring was conducted using the Way to Health (WTH) platform, an NIH-funded software platform that facilitates and automates many aspects of study design and intervention implementation.^23^

The biologic dose reminders and medication adherence check-ins were customized to each patient’s individual treatment plan. The first reminder was sent 4 days prior to the scheduled biologic dose and invoked precommitment by asking patients to reply to confirm their upcoming dose. Patients then received an additional reminder text the day before for home infusions or the day-of for self-injections. Lastly, patients were asked to confirm if they had taken their scheduled dose on the day of their scheduled self-injection or the day after their scheduled home health infusion appointment. If a patient did not confirm their scheduled dose, they received messaging that incorporated reciprocity (“we care about your health”) and anticipated regret (“we don’t want you to feel worse if you miss your dose”), and the research team followed up to help address any issues. The support partner also received messages about each upcoming dose for awareness and to help encourage adherence and provide assistance to the patient as needed.

On a weekly basis, patients were sent a brief series of prompts via text message checking in about their symptoms, utilizing the short Crohn’s Disease Activity Index (sCDAI) or the Patient Reported Ulcerative Colitis Index of Severity (PRUCIS).^10^^,^^24^ Scores and trends were monitored by the research team, and an escalation was sent to the patient’s care team through the EHR if a patient’s score reached above the pre-defined thresholds. The pre-defined thresholds as determined by clinicians were as follows: CD patient’s symptom score is ever below sCDAI = 220 and then increases above 220 OR UC patient’s PRUCIS is ever <2.5 and then increases >2.5 OR SCDAI >400 OR PRUCIS >6.

### End of study survey

At the conclusion of the 4-month study period, each patient, regardless of arm, was sent a link via text message to complete the end of study surveys online. Two additional text message reminders were sent at 3-day intervals to encourage completion of the survey if not completed within that timeframe, followed by up to 3 phone call attempts to complete the survey over the phone. The end of study survey included the SIBDQ, MARS-5, patient satisfaction survey, and one additional prompt for the intervention group only about overall satisfaction with the PATH-IBD program.

### Study outcomes

The primary outcome was the change in patient response to the SIBDQ questionnaire from baseline to end of study, as it is a validated and easy to administer way to measure changes in quality of life and disease activity.^25^ The secondary outcomes were the change in patient response to the MARS-5 and patient satisfaction questionnaires. Additional exploratory outcomes included percent days covered by biologic, net promoter score (intervention arm only), and utilization metrics including number of outpatient visits, hospitalizations, emergency department (ED) visits, and telephone/patient portal contacts. The net promoter score is computed as the percent of respondents who would likely recommend the PATH-IBD program (sore of 9 or 10 out of 10) minus the percent who are detractors (score of 0-6 out of 10).

Demographic, diagnosis, and treatment information were obtained from the EHR through automated data extraction and validated through manual chart review. Median household income was estimated using the American Community Survey 2016-2020 5-Year Estimates by zip code of residence. Medication adherence data to calculate the proportion of days covered by biologic was obtained through automated data extraction from the EHR of home health appointment information and pharmacy dispensing records and validated by manual chart review. Utilization data was obtained through automated data extraction from the EHR. Patient satisfaction, patient reported medication adherence (MARS-5) and quality of life measures (SIBDQ) were collected at the baseline and end of study surveys.

### Statistical analysis

Assuming a baseline SIBDQ score of 48.4, standard deviation of 13.5, and 15% attrition, randomizing 75 patients in each arm provided greater than 80% power to detect a 7 point increase in SIBDQ score for the between-group difference in mean change with alpha = 0.05. Randomized patients who completed the end of study survey (*N* = 144) were included in the primary and secondary analyses, and all randomized patients who completed the study (*N* = 148) were included in the exploratory analyses. Withdrawn patients were excluded from all analyses.

For the primary and secondary outcome comparisons, the Wilcoxon rank sum test was used to compare the change in SIBDQ, MARS-5, and patient satisfaction survey scores from baseline to end of study between arms, reported as median change in scores with interquartile ranges and corresponding *P-*value. The difference in medians and 95% confidence intervals was estimated through bootstrapping. For the exploratory outcomes, differences in outcomes between arms were assessed using the Wilcoxon rank sum test and the difference in medians and 95% confidence intervals were estimated through bootstrapping. Analyses were conducted using Stata version 18.0 (Stata Corp LP, College Station, Texas).

## Ethical considerations

This trial was approved by the University of Pennsylvania Institutional Review Board and granted a waiver of written consent to allow for remote recruitment as the research presented no more than minimal risk.

## Results

### Patient characteristics

**Figure 1:**
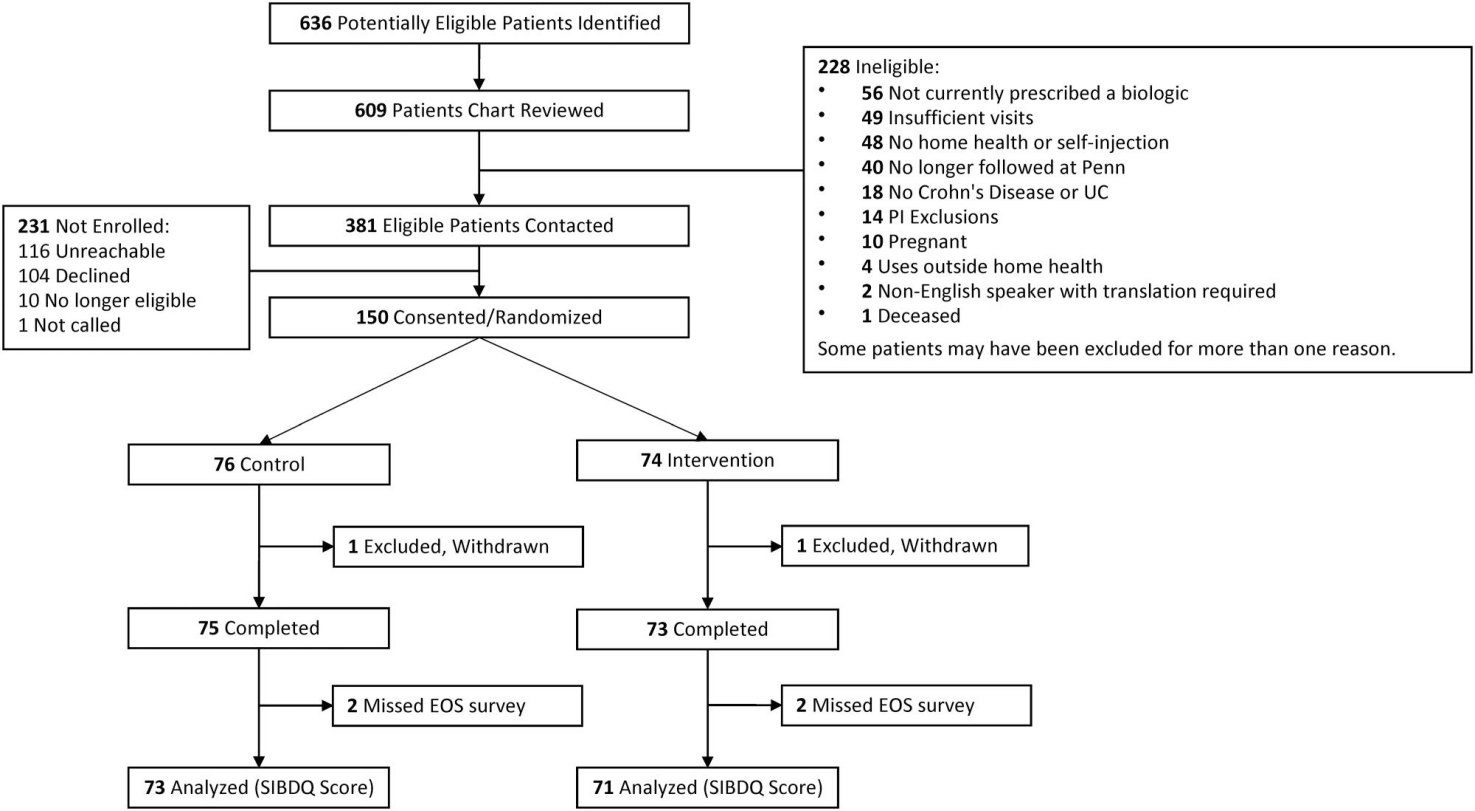
CONSORT flow diagram.

A total of 150 patients were randomized; 2 patients were withdrawn from the study due to transferring care to a new health system and, therefore, were excluded from the analysis. This resulted in 148 patients who completed the study. Additionally, 4 patients did not complete the end of study survey, resulting in 144 patients included in the primary analysis (Figure 1). No patients opted out of texting after enrolling, and all the enrolled patients received all the intended text messages. The mean age was 38.9 (SD 12.7); 52.7% were female, 81.1% were White, 9.5% were Black, and 1.4% were Hispanic or Latino; 91.2% had commercial insurance; 75.0% had Crohn’s Disease and 25.0% had Ulcerative Colitis; and 37.2% received their biologic via home health infusion while 62.8% received theirs via injection (Table 1). The intervention was conducted from March 1, 2021 through January 10, 2023, and each patient was enrolled in the trial for a total of 4 months.

**Table 1:** Patient characteristics.

	Control (*N* = 75)	Intervention (*N* = 73)	Total (*N* = 148)
**Age** ^a^			
**Mean, SD**	40.4 (13.3)	37.2 (12.0)	38.9 (12.7)
**Median, IQR**	37 (30-51)	32 (29-44)	36 (29-47)
**Age categories,** ^a^ ***N* (%)**			
**20*-*29**	16 (21.3)	25 (34.3)	41 (27.7)
**30*-*39**	27 (36.0)	21 (28.8)	48 (32.4)
**40*-*49**	11 (14.7)	14 (19.2)	25 (16.9)
**50*-*59**	15 (20.0)	7 (9.6)	22 (14.9)
**60+**	6 (8.0)	6 (8.2)	12 (8.1)
**Gender, *N* (%)**			
**Female**	41 (54.7)	37 (50.7)	78 (52.7)
**Male**	34 (45.3)	36 (49.3)	70 (47.3)
**Race, *N* (%)**			
**Asian**	4 (5.3)	3 (4.1)	7 (4.7)
**Black or African American**	5 (6.7)	9 (12.3)	14 (9.5)
**Other**^b^	1 (1.3)	2 (2.7)	3 (2.0)
**Unknown or not reported**	3 (4.0)	1 (1.4)	4 (2.7)
**White**	62 (82.7)	58 (79.5)	120 (81.1)
**Ethnicity, *N* (%)**			
**Hispanic or Latino**	0 (0)	2 (2.7)	2 (1.4)
**Not Hispanic or Latino**	75 (100)	68 (93.2)	143 (96.6)
**Unknown or not reported**	0 (0)	3 (4.1)	3 (2.0)
**Median household income by zip code,** ^c^ **median (IQR)**	$82,420 ($64,048-$105,061)	$88,858 ($62,586-$106,551)	$86,147 ($63,317-$105,806)
**Insurance type, *N* %**			
**Commercial**	70 (93.3)	65 (89.0)	135 (91.2)
**Medicare/Medicaid/TRICARE**	5 (6.7)	8 (11.0)	13 (8.8)
**Disease type, *N* (%)**			
**Crohn’s disease**	57 (76.0)	54 (74.0)	111 (75.0)
**Ulcerative colitis**	18 (24.0)	19 (26.0)	37 (25.0)
**Biologic, *N* (%)** ^d^			
**Adalimumab**	24 (32.0)	26 (35.6)	50 (33.8)
**Certolizumab**	2 (2.7)	2 (2.7)	4 (2.7)
**Golimumab**	1 (1.3)	1 (1.4)	2 (1.4)
**Infliximab**	25 (33.3)	18 (24.7)	43 (29.1)
**Ustekinumab**	18 (24.0)	19 (26.0)	37 (25.0)
**Vedolizumab**	5 (6.6)	7 (9.6)	12 (8.1)
**Biologic administration, *N* (%)** ^d^			
**Infusion**	30 (40.0)	25 (34.3)	55 (37.2)
**Injection**^e^	45 (60.0)	48 (65.7)	93 (62.8)

a Age at the time of randomization.

b Other includes Pacific Islander and Other.

c American Community Survey 2016-2020 (%-Year Estimates); in 2020 Inflation Adjusted Dollars.

d Biologic prescribed at the time of randomization.

e Includes both self-injection and home health Injection.

### Survey outcomes

The median change in SIBDQ score among patients in the intervention arm was 0 (IQR, −3 to 3) compared to −1 (IQR, −5 to 4) among patients in the control arm (difference of 1, 95% CI −0.7 to 2.7, *P* = .51), resulting in no statistically significant difference (Table 2). Both arms self-reported being fully adherent with their biologic treatment before and after the intervention, with no statistically significant difference in MARS-5 score between arms (Table 3). Overall, patients reported high satisfaction with their medical team and treatment, and there was no statistically significant change in patient satisfaction scores between baseline to end of survey between arms (Table 4). Overall, patients gave the program a Net Promoter Score of 68, as 77.4% of patients in the intervention arm gave a score of 9 or 10 out of 10 for likelihood to recommend (scale of 0-10).

**Table 2:** Change in SIBDQ score from baseline to end of study between arms.^a^

	Median baseline score, IQR	Median end of study score, IQR	Median change in score, IQR	Difference, 95% CI	*P*-Value
**Control, *N* = 73**	57 (48 to 63)	57 (45 to 63)	−1 (−5 to 4)	1 [−0.7, 2.7]	.51
**Intervention, *N* = 71**	58 (50 to 64)	57 (48 to 64)	0 (−3 to 3)		

a The SIBDQ is a 10-question instrument with the following scale: 1—All of the time, 2—Most of the time, 3—A good bit of the time, 4—Some of the time, 5—A little of the time, 6—Hardly any of the time, 7—None of the time. The composite score is reported and was calculated by summing the scores of each question.

**Table 3:** Change in MARS-5 score from baseline to end of study between arms.^a^

	Median baseline score, IQR	Median end of study score, IQR	Median change in score, IQR	Difference, 95% CI	*P*-Value
**Control, *N* = 73**	25 (24 to 25)	25 (24 to 25)	0 (−1 to 0)	0 [−0.03 to 0.03]	.13
**Intervention, *N* = 71**	25 (24 to 25)	25 (24 to 25)	0 (0 to 0)		

a The MARS-5 is a 5-question instrument with the following scale: 1—Always, 2—Often, 3—Sometimes, 4—Rarely, and 5—Never. The composite score is reported and was calculated by summing the individual scores from each question.

**Table 4:** Change in patient satisfaction scores from baseline to end of study between arms.^a^

	Control, *N* = 73			Intervention, *N* = 71			*P*-Value
	Median baseline score, IQR	Median End of study score, IQR	Median change in score, IQR	Median baseline score, IQR	Median End of study score, IQR	Median change in score, IQR	
**I feel connected to my medical team. **	2 (1 to 2)	2 (1 to 2)	0 (0 to 0)	2 (1 to 2)	2 (1 to 2)	0 (0 to 0)	.17
**I feel my medical team is aware of changes in my condition.**	2 (1 to 2)	2 (1 to 2)	0 (0 to 1)	2 (1 to 2)	2 (1 to 2)	0 (−1 to 0)	.06
**I feel like I’m on track with my treatment plan.**	2 (1 to 2)	2 (1 to 2)	0 (0 to 1)	2 (1 to 2)	2 (1 to 2)	0 (−1 to 0)	.12
**The medical personnel who look after me know my medical history and concern themselves with the evolution of my bowel disease.**	2 (1 to 2)	2 (1 to 2)	0 (0 to 1)	1 (1 to 2)	1 (1 to 2)	0 (0 to 0)	.21
**The staff that look after me and the place I go for treatment motivate me to stick with the treatment for my illness.**	2 (1 to 2)	2 (1 to 3)	0 (0 to 1)	2 (1 to 2)	1 (1 to 2)	0 (0 to 0)	.29
**If any problems arise with the treatment I am receiving, my medical team resolve it quickly and effectively.**	2 (1 to 2)	2 (1 to 2)	0 (0 to 1)	2 (1 to 2)	2 (1 to 2)	0 (0 to 0)	.48

a Scale: 1—Totally Agree, 2—Agree, 3—Neither Agree or Disagree, 4—Disagree, 5—Totally Disagree.

### Proportion of days covered and utilization

Overall, patients had high adherence, and there was no statistically significant difference in the proportion of days covered for infusion (difference of 0, 95% CI −2.0 to 2.0, *P* = .59) or injection (difference of −2.9, 95% CI −8.0 to 2.2, *P* = .51) patients between arms (Table 5). In the intervention group, there was high self-reported adherence to their IBD medication during the text check-ins, with a mean 88.5% adherence (standard deviation 17.4) and median 100% adherence (interquartile range 76.9 to 100). There was no statistically significant difference in outpatient visits, hospitalizations, or ED visits between arms; however, there was statistically greater GI telephone/patient portal contacts in the intervention arm. Patients in the intervention arm had a median total of 5 GI contacts during the intervention period, while the control arm had a median total of 3 GI contacts (difference of 2, 95% CI 0.4 to 3.6, *P* < .001) (Table 6). Weekly sCDAI and PRUCIS score trajectories for the intervention group are shown in Supplement Figures S1 and S2. In the intervention group, there were 38 escalations to the clinical team among 26 unique patients, which resulted in outreach by the nurse to the patient via phone call or patient portal message.

**Table 5:** Proportion of days covered between arms among infusion and injection patients.

	Arm	Median, IQR	Difference, 95% CI	*P*-Value
**Proportion days covered, infusion^a^**	Control, *N* = 30	100 (97.5 to 100)	0 [−2.0 to 2.0]	.59
	Intervention, *N* = 25	100 (95 to 100)		
**Proportion days covered, injection^b^^,^^c^**	Control, *N* = 45	100 (88.3 to 100)	−1.7 [−6.7 to 3.3]	.57
	Intervention, *N* = 43^d^	98.3 (90 to 100)		

a Determined based on Home Health infusion appointment dates and administration data.

b Determined using pharmacy refill data.

c One patient received injection through Home Health, proportion days covered calculated using Home Health appointment data.

d Pharmacy refill data unavailable for 6 patients.

**Table 6:** Comparison of health care utilization metrics between arms.

	Median, IQR		Difference, 95% CI	*P*-Value
	Control, *N* = 75	Intervention, *N* = 73		
**All outpatient visits**	2 (0 to 4)	2 (1 to 5)	0 [−1 to 1]	.64
**GI outpatient visits**	1 (0 to 1)	1 (0 to 1)	0 [−1.2 to 1.2]	.76
**Hospitalizations**	0 (0 to 0)	0 (0 to 0)	0 [N/A]	.33
**ED visits**	0 (0 to 0)	0 (0 to 0)	0 [N/A]	.10
**All contacts**	5 (2 to 8)	7 (4 to 12)	2 [−0.3 to 4.3]	.007
**GI contacts**	3 (1 to 6)	5 (3 to 8)	2 [0.4 to 3.6]	<.001

## Discussion

In this trial of remote text monitoring for patients with IBD on biologic therapy, the intervention did not result in greater SIBDQ score, medication adherence, or patient satisfaction. The intervention was feasible and resulted in greater communication from the patient via the patient portal, but no other differences in outpatient, ED, or hospital visits were found.

We hypothesize several possible reasons for the lack of effect of remote monitoring on quality of life or medication adherence. The research consent process may have selected for more highly motivated patients. Both the control and intervention groups had high baseline SIBDQ scores and almost perfect medication adherence based on self-report and EHR data. As such, there was little room for improvement in either of these metrics. Moreover, since most of the patient population was on a stable regimen for their IBD, it is possible that the 4-month intervention period may not have been long enough to alter care for patients, and the sample size may not have been large enough to detect smaller differences. Due to capacity constraints of the IBD clinic during the COVID-19 pandemic, we had to use a high threshold for escalation to care team. Finally, more intensive engagement with clinicians may be needed to improve IBD-related clinical outcomes.

A large 12-month trial of 909 patients in the Netherlands evaluating a web-based monitoring and engagement program showed reduction in outpatient visits and hospitalizations, increase in medication adherence, but no changes in quality of care scores.^16^ Another 12-month trial of 348 patients in the US evaluated a text-based monitoring program with dedicated nursing staff, showing no improvement in quality of life or disease activity, but reduced IBD-related hospitalizations.^17^ Our trial was of shorter duration and relied on existing clinical staff who did not have as much capacity to engage with patients.

Despite the lack of superiority of the intervention over usual care, it was well received by the participants. The net promotor score was 68, with more than three quarters of the participants being likely to recommend PATH-IBD. While we are not able to identify the features of PATH-IBD that participants most valued, the PATH-IBD arm had a greater number of patient contacts, reflecting closer ties with the practice, one of the goals of the intervention. Given that providers caring for patients with IBD already spend more time in the electronic health record than most other gastroenterologists and often express dissatisfaction with the electronic health record, some providers may view remote contact programs such as PATH-IBD as an added burden.^26^^,^^27^ However, the majority of the contacts were able to be handled by nurses.

The strengths of this study are the use of user-friendly text messaging to engage with patients and integration into routine clinical practice for care escalation. We also used behaviorally-informed messaging including concepts of precommitment, reciprocity, and social support, which resulted in high engagement by patients. There were also some limitations. The cohort of patients at an urban academic gastroenterology program may not translate to other clinical environments. We were able to measure patient-reported outcomes, medication adherence, and utilization but could not evaluate more objective clinical markers due to the pragmatic nature. We were only able to measure outcomes for 4 months, so it may take a longer intervention to demonstrate effectiveness. Also, the opt-in research consent process may have selected a patient group not fully representative of the general population.

## Conclusion

We show that a remote text monitoring program for IBD is feasible with high patient satisfaction, but it did not result in improvements in quality of life or medication adherence in a cohort of patients who already demonstrated high adherence and IBD-related quality of life. Future studies could evaluate a more personalized intervention targeting patients at risk for poor adherence or with low self-efficacy related to their IBD management, dedicated staffing, lower thresholds for escalation, and longer-term outcomes across a broader patient population.

## Supplementary Material

otag076_Supplementary_Data

## Data Availability

Data is not publicly available for this study, since we do not have approval from our institution.
