# Supplementary material for: Remote monitoring to improve engagement for patients with inflammatory bowel disease: a randomized clinical trial
Source: Crohns Colitis 360. 2026 Jul 7;8(3):otag076. doi: 10.1093/crocol/otag076 (PMC13384052; doi:10.1093/crocol/otag076)

**Supplemental Materials**

Supplement Figure 1. Weekly short Crohn’s Disease Activity Index (sCDAI) score in intervention group


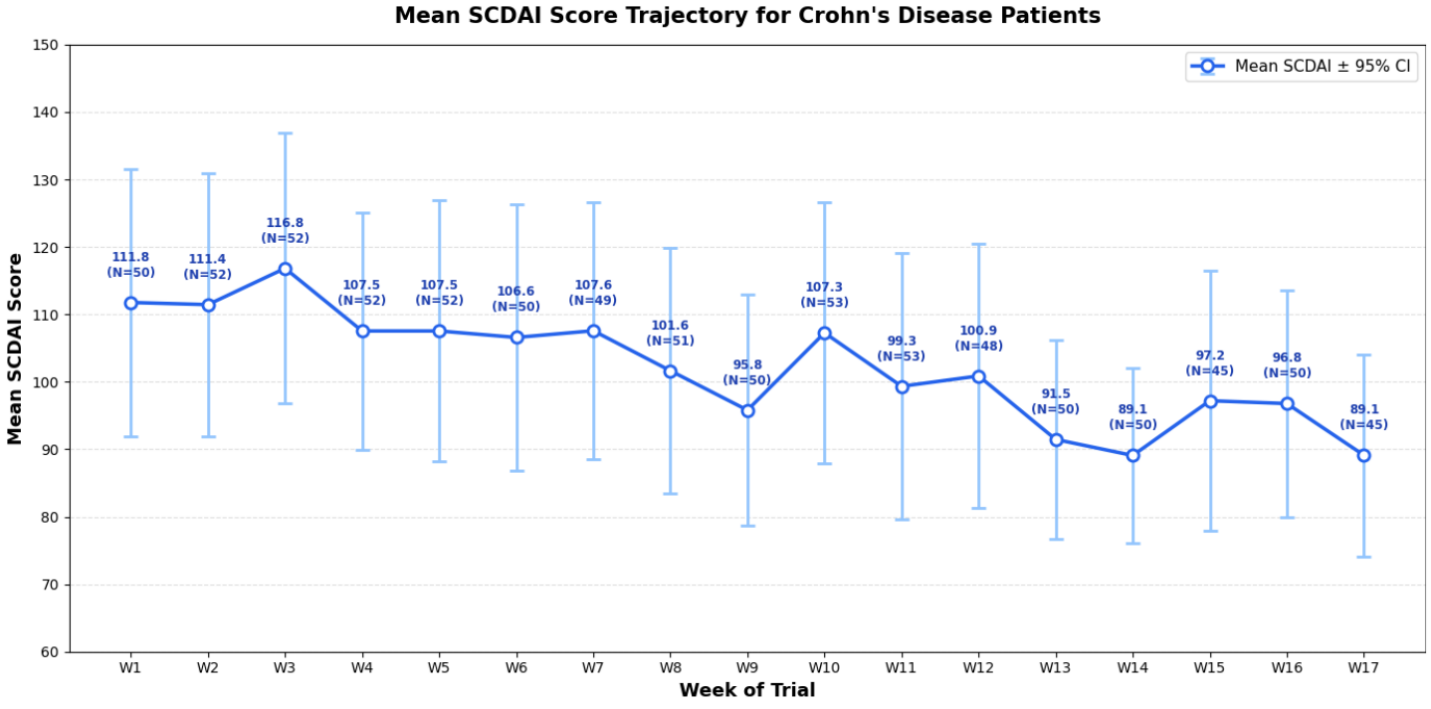


Supplement Figure 2. Weekly Patient Reported Ulcerative Colitis Index of Severity (PRUCIS) score in intervention group


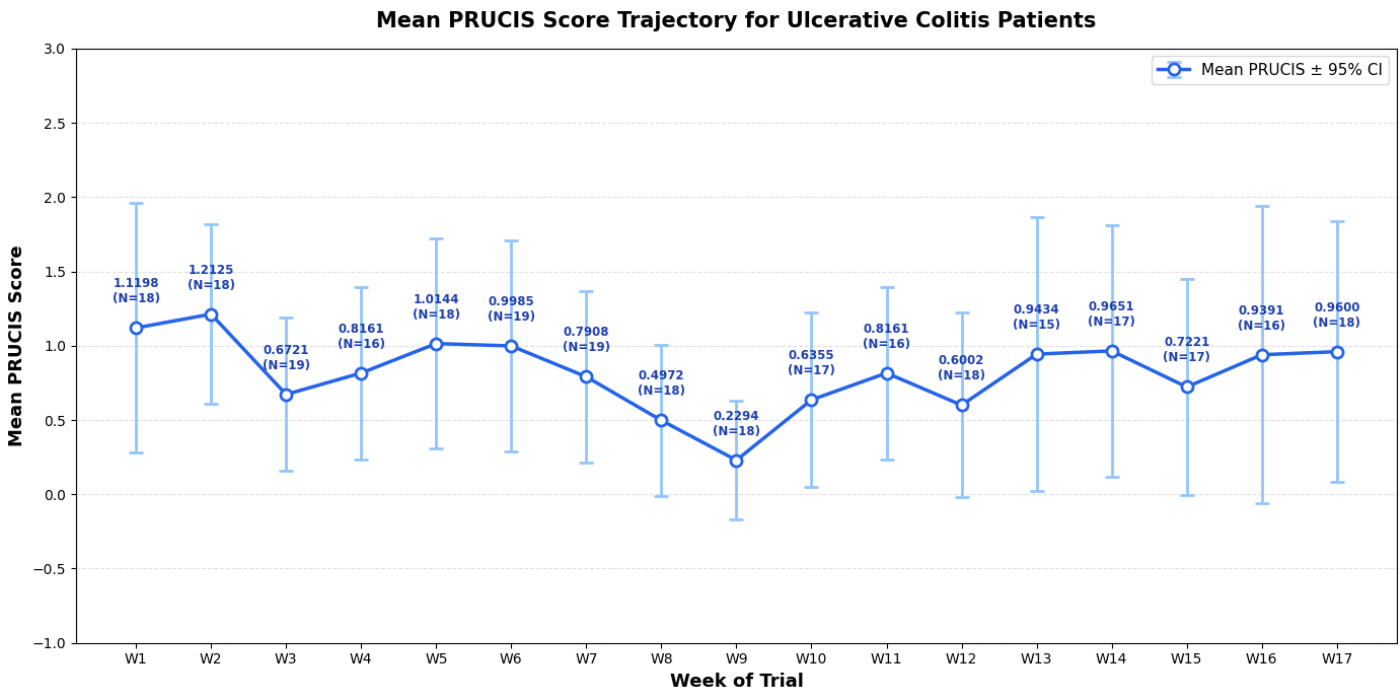

Supplement: otag076_Supplementary_Data [file otag076_supplementary_data.docx]
